# Influence of Simvastatin-Loaded Implants on Osseointegration in an Ovariectomized Animal Model

**DOI:** 10.1155/2015/831504

**Published:** 2015-03-29

**Authors:** Wen Fang, Shifang Zhao, Fuming He, Li Liu, Guoli Yang

**Affiliations:** ^1^School of Medicine, Zhejiang University, Yan'an Road, Hangzhou 310000, China; ^2^Department of Implantology, Stomatology Hospital, School of Medicine, Zhejiang University, Yan'an Road, Hangzhou 310000, China; ^3^Department of Prosthodontics, Stomatology Hospital, School of Medicine, Zhejiang University, Yan'an Road, Hangzhou 310000, China

## Abstract

The success of bone implants in the presence of osteoporosis is limited by lack of osseointegration between the implant and the natural bone. This study applied an electrochemical process to deposit simvastatin-nanohydroxyapatite (HA) coatings on porous implant surfaces and investigated the effects of these simvastatin-HA coatings on implant surfaces in an animal model of osteoporosis. In this study, simvastatin-HA coated implants were inserted into the tibia of osteoporotic rats. After 2, 4, and 12 weeks, tissue was retrieved for histomorphometric evaluation. The results indicated that the simvastatin-HA coatings increased bone-implant contact and new bone formation around implant surfaces. In conclusion, implants loaded with simvastatin by an electrochemical process improved implant osseointegration in osteoporotic rats. Furthermore, the increased concentration of simvastatin could affect the osseointegration, but the dose-effects also need further investigation.

## 1. Introduction

Osteoporosis is an increasing health problem. Reduced bone strength is the main characteristic of osteoporosis, resulting in an increased risk of fractures [1]. Studies show that osteoporosis increases bone resorption around the teeth or in the edentulous ridge [2]. However, no direct link has been shown between osteoporosis and implant failure [3]. Bone-to-implant contact decreases because of the decrease in bone mass. This results in the reduction of support strength of implant. Therefore, the focus of this study is how to improve implant osseointegration in the presence of osteoporosis.

To date, bisphosphonates have been the main class of drug used to improve implant osseointegration in osteoporosis [4]. Research has shown that bisphosphonates increase bone-implant contact and bone-bonding strength. However, recent investigations showed that jaw necrosis can occur with the administration of bisphosphonates, which is a safety concern. Moreover, bisphosphonates are mainly antiresorptive agents, which are inhibitors of bone resorption acting mainly to stabilize bone mass and prevent further bone loss. Their effect on increasing the bone mass is therefore modest [5].

The 3-hydroxy-3-methylglutaryl-coenzyme A reductase inhibitors, known as statins, are widely used cholesterol-lowering drugs which inhibit hepatic cholesterol biosynthesis. Recent studies showed beneficial effects of statins on bone mineral density [6, 7]. Simvastatin, a liposoluble statin, induces the expression of bone morphogenetic protein (BMP)-2 mRNA resulting in bone formation on the calvaria of mice following daily subcutaneous injections [8]. There are more investigations on statin metabolic effect. For instance, simvastatin improved cancellous bone mass and bone compressive strength by oral administration [9]. Ayukawa et al. [10] confirmed that new bone tissue increased by topical application of statins. In addition, bone mineral density also increased using of statin by clinical investigations [11, 12].

Most literature reports refer to systemic administration of simvastatin. However, when statins were topically applied or continuously released from an implant, they were 50–80 times more effective in inducing bone formation than when given perorally or injected subcutaneously [13]. We have applied simvastatin onto implant surfaces and found it improved osteoblast function and implant osseointegration in osteoporosis [14, 15]. However, we also found simvastatin was immediately released from implant surfaces and the half-life of the drug was short [16]. An optimal carrier for simvastatin would slowly release the drug in the carrier solution. This approach promises to improve the osseointegration of the implant.

We used an electrochemical process to deposit a hydroxyapatite (HA) coating onto porous implant surfaces. These HA crystals were rod-like with a hexagonal cross section. In vivo experiments showed this HA coating increased bone formation around the implant and implant stability [17]. The electrochemical process was also used to deposit simvastatin onto the implant surface. In vitro experiments demonstrated that a simvastatin-HA coating was formed on the porous implant surface and improved osteoblast function [18]. An LC-MS/MS test showed the effective simvastatin concentration was 1.89 × 10^−7^ to 2.77 × 10^−7^ mol/L/cm^2^. The aim of this study was to investigate the effects of a simvastatin-HA coating on bone integration with implant surfaces in an animal model of osteoporosis.

## 2. Materials and Methods

### 2.1. Titanium Implants and Surface Treatments

Surface treatment of implants (*n* = 72) was similar to the previous study [8]. The diameter of implant was 2.2 mm and the length was 4.0 mm. Samples were treated by large corundum grit blasting, HF/HNO_3_ solution, and HCl/H_2_SO_4_.

### 2.2. Preparation of Simvastatin Solution

Simvastatin solution was prepared as previously described [18]. In brief, 42 mg simvastatin was dissolved in 1 mL of 95% ethanol, and 1.5 mL of 0.1 M NaOH was added. Then, the solution was neutralized to pH 7.2 with 0.1 M HCl, and the volume was brought to 10 mL with deionized water. Simvastatin acid (10 mM) was prepared.

### 2.3. Preparation of HA and Simvastatin-HA Coatings

The preparation of HA coatings (control group) was as described in previous studies [18]. The working electrode (cathode) was implant and the counter electrode was a platinum (Pt) plate. The electrolytes were prepared by dissolving analytical grade Ca(NO_3_)_2_ (0.6 mM) and NH_4_H_2_PO_4_ (0.36 mM) into distilled water in a Ca/P ratio of 1.67. NaNO_3_ (0.1 M) was added to improve the conductivity of the electrolytes. The deposition process was conducted with a DC power source at 3.0 V at 85°C for 30 min. Preparation of simvastatin-HA (Sim-HA) coatings used the same process except that simvastatin was added to the electrolyte solution in varying concentrations from 10^−7^ M (test 1 group) to 10^−6^ M (test 2 group). A field-emission scanning electron microscopy (FSEM, FEI, SIRION100) was used to perform surface morphology.

### 2.4. Animal Experiments

Experiments were approved by the Institutional Animal Care and Use Committee of Zhejiang University, Hangzhou, China. Thirty-six female Sprague-Dawley rats (250–350 g) were used in this study. Surgery was performed under sterile conditions in a veterinary operating theatre. All of the 36 rats were divided into three groups, including test 1, test 2, and control group (12 rats per group). Four rats were euthanized at 2, 4, and 12 weeks, respectively, for every group.

Implantation was performed as described in previous studies [19, 20]. Implantation for the establishment of the standard osteoporotic animal model was performed at 12 weeks after bilateral ovariectomy. Two different implants were inserted into the distal tibia of one rat. 10% chloral hydrate (0.35 mL/100 g, i.m.) was applied for systemic anesthesia. Lidocaine was applied on surgical site. One osteotomy with the diameter of 2.2 mm was prepared under drilling with a low speed and refrigeration. Implants were inserted into the osteotomy. Antibiotics (Penicillin, 400,000 U/d) were applied for 3 days.

Animals were euthanized by using an overdose of chloral hydrate at 2, 4, and 12 weeks following the operation. Tissue was retrieved for histomorphometric evaluation.

### 2.5. Specimen Preparation and Histomorphometric Evaluation

Specimen preparation was according to previous method [15]. The tibias containing the implants were fixed in 4% neutral-buffered formaldehyde, dehydrated, and embedded in methyl methacrylate. Undecalcified cut and ground sections containing the central part of the implants were produced at a final thickness of 30 *μ*m using a Macro cutting and grinding system (Exakt 310 CP series, Exakt Apparatebau, Norderstedt, Germany). The sections were stained with Stevenel's blue and van Gieson's picro fuchsin. Light microscopy (BX51, Olympus, Japan) and a PC-based image analysis system (Image-Pro PlusR, Media Cybernetics, Silver Springs, MD, USA) were used for histometric analysis. The percentage of bone-to-implant contact (BIC) in the threads along the total length of the implant surface and the percentage of bone area inside the same threads were measured.

### 2.6. Statistical Analysis

Histomorphometric data were analyzed statistically. Group means and standard deviations were calculated for each parameter. Differences between experimental samples were analyzed by one-way analysis of variance with post hoc tests. A *P* value < 0.05 was set as the threshold for statistical significance. SPSS version 16.0 software (SPSS) was used for all statistical analyses.

## 3. Results

### 3.1. Surface Analysis

**Figure 1:**
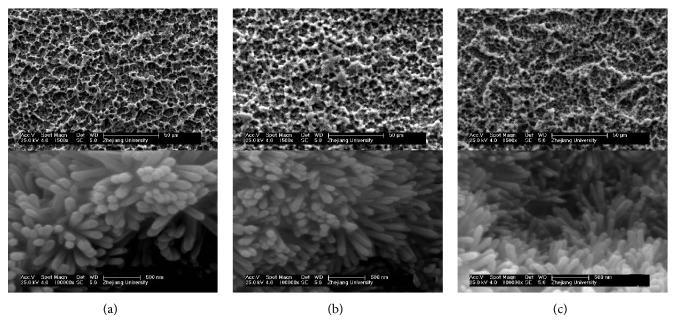
FSEM observation of control and test implant surfaces. (a) Test 1 group. (b) Test 2 group. (c) Control group.

Similar topography appeared on coatings of implant surfaces in FSEM observation (Figure 1). Porous topography was still clear on three implant surfaces. Most crystals were rod-like with a hexagonal cross section, which covered the irregular surfaces. These indicated the coatings were thin. However, drug seemed to affect the size of hydroxyapatite crystals. Simvastatin-HA crystals (test 1 and test 2, Figures 1(a) and 1(b)) were smaller than HA crystals (control, Figure 1(c)). Meanwhile, there were no differences for the crystals between control group and sham group (only performing the surgery operation, data not shown). And the shape of crystals became irregular. The end of crystals became sharp. The previous studies showed the crystals became small with the increase of drug concentration.

### 3.2. In Vivo Experiments

After operation, rats quickly survived and recovered. The rats appeared to be in good health throughout the test period. At sacrifice, there were no clinical signs of inflammation or adverse tissue reactions. Samples remained in situ at sacrifice.

### 3.3. Histologic Findings

**Figure 2:**
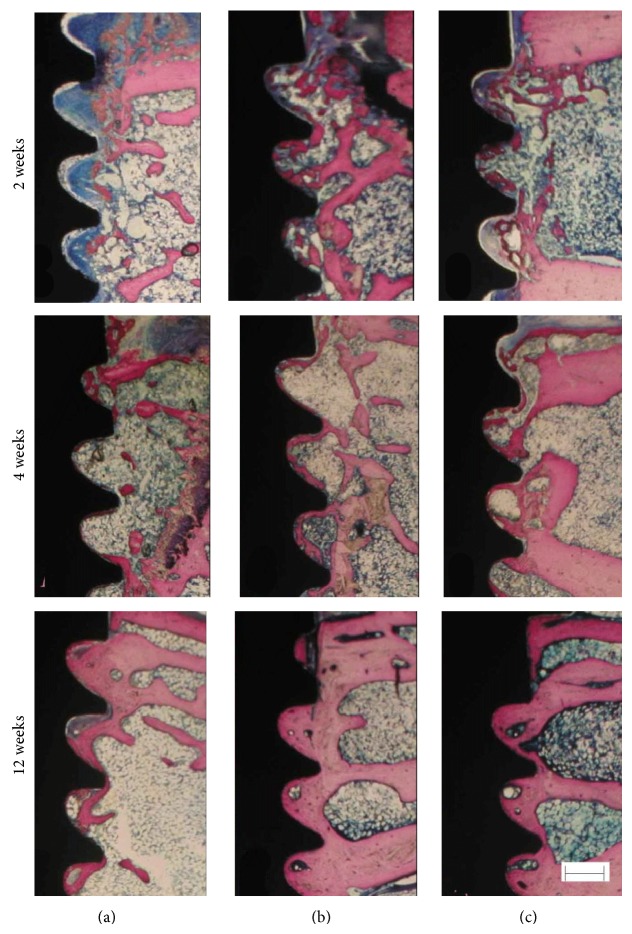
Histologic observation of control and test groups after 2, 4, and 12 weeks. (a) Control group. (b) Test 1 group. (c) Test 2 group. Bar = 320 *μ*m.

After 2 weeks, there was little new bone around control implant surfaces. The bone tissue was not in direct contact with implant surface. Around test 1 and 2 implant surfaces, woven bone tissue appeared, which was in direct contact with implant surfaces as shown in Figure 2. After 4 and 12 weeks, new bone had matured around control and test implant surfaces as shown in Figure 2. New bone had direct contact with implant surfaces. There was more bone tissue around test implant surfaces. More bone-implant contact also appeared on test implant surfaces. Also, there were no differences between the control and sham group (data not shown).

**Figure 3:**
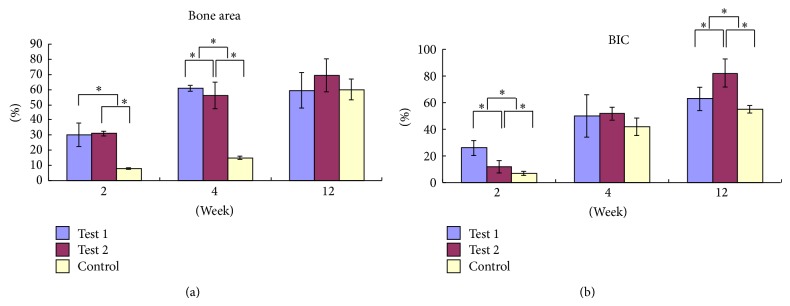
The mean bone area percentage within all threads and BIC with all threads along the total length of the implant surface. (a) Bone area percentage. (b) BIC.

The means of the bone area percentage within all threads are shown in Figure 3(a). After 2 weeks, there were significant differences between test groups and control group (*P* < 0.01, *P* < 0.01, resp.). No differences were found between test 1 group and test 2 group (*P* > 0.05). After 4 weeks, there were clear differences between the test groups and control group (*P* < 0.01, *P* < 0.01, resp.) and between test 1 group and test 2 group (*P* < 0.05). After 12 weeks, there were no clear differences between the test groups and control group (*P* > 0.05, *P* > 0.05, resp.) or between test 1 group and test 2 group (*P* > 0.05). Among the control group, there were significant differences in bone area between 2 weeks and 4 weeks (*P* < 0.05), between 2 weeks and 12 weeks (*P* = 0.000), and between 4 weeks and 12 weeks (*P* < 0.01). Among test 1 group, significant differences were found between 2 weeks and 4 weeks (*P* < 0.01) and between 2 weeks and 12 weeks (*P* < 0.01). No significant differences were found between 4 weeks and 12 weeks (*P* > 0.05). Among the test 2 group, there were significant differences in bone area between 2 weeks and 4 weeks (*P* < 0.01), between 2 weeks and 12 weeks (*P* < 0.01), and between 4 weeks and 12 weeks (*P* < 0.05).

BIC with all threads along the total length of the implant surface is shown in Figure 3(b). After 2 weeks, there were significant differences between the test groups and the control group (Figure 3(b), *P* = 0.000, 0.048, resp.) and between test 1 group and test 2 group (*P* < 0.01). After 4 weeks, no significant differences were found between the test groups and control group (*P* > 0.05, *P* > 0.05, resp.) or between test 1 group and test 2 group (*P* > 0.05). After 12 weeks, there were significant differences between the test groups and control group (*P* < 0.05, *P* < 0.01, resp.) and between test 1 group and test 2 group (*P* < 0.001). In the control group, there were significant differences in BIC between 2 weeks and 4 weeks (*P* < 0.01), between 2 weeks and 12 weeks (*P* < 0.01), and between 4 weeks and 12 weeks (*P* < 0.01). In test 1 group, there were significant differences in BIC between 2 weeks and 4 weeks (*P* < 0.01), between 2 weeks and 12 weeks (*P* < 0.01), and between 4 weeks and 12 weeks (*P* < 0.05). In test 2 group, there were significant differences in bone area between 2 weeks and 4 weeks (*P* < 0.01), between 2 weeks and 12 weeks (*P* < 0.01), and between 4 weeks and 12 weeks (*P* < 0.01).

Furthermore, there were no differences for the bone area percentage and BIC within all threads between control group and sham group (data not shown).

## 4. Discussion

This study investigated the effect of a simvastatin-HA coating on implant osseointegration in an osteoporosis animal model. The results showed the simvastatin-HA coating improved the bone-implant contact and new bone formation around implant in the osteoporotic rats.

Our findings were similar to those of earlier reports on the osteogenic effects of statins. Mundy et al. [7] found the positive function of simvastatin on bone tissue. Then, there were more investigations on statins. Systemic administration was described in many studies, including oral and intraperitoneal administration. Investigations also showed that systemic administration improved osseointegration of pure titanium implants in normal or osteoporotic rats [21, 22]. Recently, local administration of drugs which have the potential to improve osseointegration has been the focus of research. Nyan et al. [23] applied simvastatin onto porous implant surfaces by wetting homogenously and also demonstrated clear effects on implant osseointegration. Although the local administration methods and animal models were different, the results were similar.

Our results indicated that the simvastatin treatment affected the size of hydroxyapatite crystals in test 1 and test 2 groups significantly. However, no changes were discovered in the control group. These results suggested that the simvastatin could improve the topography on coatings of implants in FSEM observation. The histologic findings indicated that more bone tissues around test implant surfaces and more bone-implant contact appeared in test groups. The results suggested that the simvastatin treatment enhance the bone-implant contact for the ovariectomized animal model.

In order to confirm the above results, we also examined the bone area percentage within all threads and the BIC with all threads along the total length of the implant surface. The results showed that the simvastatin treatment could totally increase the bone area percentage and the BIC levels. Interestingly, the bone area percentage in test 1 and test 2 group was significantly enhanced compared to the control group at 2 and 4 weeks; however, no differences were found at 12 weeks. This result suggested that the effects of simvastatin-loaded implants on bone area formation mainly occurred before 12 weeks, and also the simvastatin-loaded implants could shorten the cure time of the bone injury at early time.

The detailed mechanism of action of the stimulatory effect of simvastatin on bone formation is not well understood. BMP-2 expression was increased by simvastatin, which subsequently improved synthesis of differentiation markers characteristic and suppressed gene expression of matrix metalloproteinase 13 (collagenase-3) [24]. Nitric oxide production through statin-induced upregulated endothelial nitric oxide synthase also plays key roles in regulating osteoblast differentiation and bone formation [25, 26]. Furthermore calcyclin, a Ca^2+^ ion-binding protein, was identified by proteomic analysis to be significantly upregulated when treated with simvastatin and it has been suggested that this protein plays an important role in the anabolic effect of simvastatin on bone [27].

Another reason may be the suppression of simvastatin on osteoclast differentiation through the inhibition of the gene* Src* and the receptor activator of nuclear factor kappa B ligand as well as the enhancement of mitogen-activated protein kinase/Akt pathways [10, 28]. Osteoporosis results in the presence of more osteoclasts in bone tissue. Therefore, suppression of osteoclast differentiation by simvastatin is important for implant integration with bone.

Simvastatin is released slowly in a solution of HA, which promotes implant osseointegration. Two types of binding of simvastatin with HA crystals were noted in this study, namely, physical absorption and chemical bonding. Physical adsorption of simvastatin results in instantaneous release of simvastatin while the chemical bonding of simvastatin results in its gradual release with the dissolution of HA. Both types of binding were effective in improving osteoblast function. In vitro experiments also demonstrated the incorporation of simvastatin into HA crystals at the time of its deposition and its influence on the size of HA crystals. Further research is necessary into the way that simvastatin bonds chemically with HA.

Many different carriers of simvastatin have been described in the literature, including sequential platelet-derived growth factor-simvastatin delivery [29], simvastatin gel [30], a blend of cellulose acetate phthalate and a poly(ethylene oxide) and poly(propylene oxide) block copolymer [31], simvastatin-containing polycaprolactone scaffolds [32], and a mixture of lactic acid/glycolic acid copolymer, alpha-tricalcium phosphate, and calcium carbonate [33]. In this study, we reported the electrochemical coating method, which indicated a lot of advantages compared with the previous reported methods [29–33]. The previous carriers of simvastatin always trigger some disadvantages, such as different side-effects, delayed therapeutic effects, or the high-priced materials. Therefore, we hold the view that the present method is noninvasive, cheap, and convenient, which may be used in the clinic.

In this study, we used an electrochemical process to assemble simvastatin onto implant surfaces. This electrochemical process deposited a nano-HA coating on implant surfaces, in a way that is similar to the structure of HA in bone. The porous topography of implant surfaces was still clear because the coatings were very thin. These assemblies were suitable for clinical application.

## 5. Conclusions

It was concluded that simvastatin-loaded implant surfaces by the electrochemical process improved implant osseointegration in osteoporosis rats. This opens up the possibility of better clinical application of implants in the presence of osteoporosis in humans. Furthermore, the increased concentration of simvastatin could affect the osseointegration, but the dose-effects also need further investigation.
